# The influence of pain catastrophizing and central sensitization on the reported pain after hip arthroscopy

**DOI:** 10.1007/s00167-021-06658-w

**Published:** 2021-07-11

**Authors:** Niels H. Bech, Inger N. Sierevelt, Aleid de Rooij, Gino M. M. J. Kerkhoffs, Daniel Haverkamp

**Affiliations:** 1 Specialized Center of Orthopaedic Research and Education (SCORE), Department of Orthopedic Surgery, Xpert Orthopedic Surgery Clinic, Amsterdam, The Netherlands; 2 Department of Orthopaedic Surgery, Amsterdam UMC, University of Amsterdam, Amsterdam Movement Sciences, Amsterdam, The Netherlands; 3 Centre for Orthopaedic Research, Spaarne Ziekenhuis, Hoofddorp, The Netherlands; 4 Amsterdam Rehabilitation Reserach Centre, Reade, Amsterdam, The Netherlands; 5 Academic Center for Evidence-Based Sports Medicine (ACES), Amsterdam, The Netherlands; 6 Amsterdam Collaboration on Health and Safety in Sports (ACHSS) AMC/Vumc, IOC Research Center, Amsterdam, The Netherlands

**Keywords:** Pain catastrophizing, Central sensitization, Hip arthroscopy, Pain

## Abstract

**Purpose:**

This study was conducted to investigate whether the pain catastrophizing scale (PCS) and the central sensitization inventory (CSI) are predictive factors for the reported pain after hip arthroscopy.

**Methods:**

A total of 37 patients undergoing hip arthroscopy for femoroacetabular impingement syndrome and labral tears were prospectively enrolled. All patients completed the PCS and CSI before hip arthroscopy. Postoperative pain was measured with the numeric rating scale (NRS) weekly the first 12 weeks after surgery by electronic diary.

**Results:**

At baseline, univariate analyses showed that both the CSI and PCS were significantly associated with the NRS outcome (*p* < 0.01). During 12 weeks follow-up, a significant decrease on the NRS was observed (*p* < 0.01). Univariate analyses showed that both the CSI and PCS were significantly associated with the NRS during follow-up. Multivariate mixed model analysis showed that only the PCS remained significantly associated with the NRS outcome with a ß of 0.07 (95% CI 0.03–0.11, *p* < 0.01).

**Conclusion:**

Results indicate that both the PCS and CSI are associated with the reported postoperative pain after hip arthroscopy. The PCS and CSI may be useful in daily practice to identify patients that possibly benefit from pain catastrophizing reduction therapy (e.g. counseling) prior to surgery.

**Level of evidence:**

IV

## Introduction

Postoperative pain after hip arthroscopy is usually measured as numeric rating scale (NRS) and is commonly used as outcome after surgery [17]. Although measuring of postoperative pain with the NRS is well validated there are several patient-related factors that might influence the reported postoperative NRS score. In current literature, several factors are described as being risk factors for negative outcome after surgery, for example: female gender, increasing age, duration of symptoms before surgery, presence of pre-operative osteoarthritis and an increased BMI [3, 16, 28].

Other non-orthopedic factors, such as patient mental health and psychological state, might influence the reported postoperative pain as well [10, 12, 27]. One of those factors might be central sensitization (CS) in which an abnormal enhancement of the pain mechanism may be present involving the central nervous system [20, 22]. Central sensitization is defined as an increased sensitivity of the central nervous system [5]. Central nervous system hyper-excitability is associated with various symptoms for example pain. Basically, it can be said that processing of nociceptive inputs can differ between individuals resulting in a different perception of pain [20, 22]. For example, CS is a reported risk factor for persistent pain, patient dissatisfaction and lower quality of life in patients undergoing total knee arthroplasty [15]. For measuring symptoms related to CS the central sensitization inventory (CSI) is used and a cut-off value of 40 out of 100 points is determined to identify patients with central sensitization syndrome (CSS) [19, 21, 22].

Another non-surgical factor that may be of influence on the reported postoperative pain is pain catastrophizing (PC). If PC is present, the patient has a tendency to magnify the threat value of a pain stimulus and to feel helpless in the presence of pain, also controlling pain-related thoughts can be a problem [24, 29]. PC has shown to be related to higher levels of pain and suffering and worse outcome after musculoskeletal surgery [1, 8, 13, 23]. PC is usually measured and validated with the pain catastrophizing scale (PCS) [29].

If factors, such as PC and CS, play a role in postoperative pain there might be a reason for routine pre-operative measuring both scores. The aim of this study is to investigate the role of PC and CS on the reported pain after hip arthroscopy and the hypothesis is that both CS and PC are of influence on the reported pain after hip arthroscopy.

## Materials and methods

All included patients were part of a trial for which the study protocol was approved by the medical ethical committee (NL55669.048.15). Inclusion criteria for our current study were a confirmed diagnosis of Femoroacetabular Impingement Syndrome (FAIS), age between 18 and 65 years and a completed CSI and PCS. FAIS is considered abutment of the proximal femur to the acetabular rim [2]. Diagnosis of FAIS was made with plain radiographs and MRI by measuring the alpha angle, lateral center edge angle and measuring a possible cross-over sign. Exclusion criteria were previous hip arthroscopy or hip surgery, indications for hip arthroscopy other than FAIS and/or a BMI > 35.

All patients were operated by a single orthopedic surgeon (D.H) with good hip arthroscopy experience (> 1000 procedures performed and > 150 annually). Procedures were performed in either a general hospital or a private orthopedic clinic. A total of 37 patients completed both the PCS and CSI and were included in our current study. Baseline characteristics are shown in Table 1.

**Table 1:** Baseline and clinical characteristics (*n* = 37)

Demographics	
Age (years), mean (SD)	35.4 (10.4)
BMI, mean (SD)	23.6 (2.8)
Gender, *n* (%)	
Male	23 (62)
Female	14 (38)
Operation details	
CAM, *n* (%)	15 (41)
Pincer, *n* (%)	20 (54)
Labral repair, *n* (%)	20 (54)
Psoas lengthening, *n* (%)	2 (5)
PROMs	
NRS_pain_, mean (SD)	4,0 (2.5)
CSI, mean (SD)	30.5 (17.1)
PCS, mean (SD)	16.6 (11.3)

*NRS* numeric rating scale, *CSI* central sensitization inventory, *PCS* pain catastrophizing scale, *SD* standard deviation

### Outcome

Pain was measured using a Numeric Rating Scale (NRS pain) and all patients were asked to complete a Central Sensitization Inventory (CSI) and a Pain Catastrophizing Scale (PCS) before surgery. The NRS was measured pre-operatively at baseline and weekly after surgery until 12 weeks post-operatively (by electronic diary). The outcome ranges between 0 and 10 where 0 means no pain and 10 worst possible pain. The NRS is a validated tool for measuring pain [6].

The CSI is a validated tool that is used to identify patients who have symptoms that may be related to CS [19]. The questionnaire consists of 25 questions and a score between 0 and 100 (best to worst) can be reached. A score of more than 40 indicates the presence of central sensitization [20].

PC was measured with the pain catastrophizing scale (PCS). The questionnaire which measures three components of pain catastrophizing being rumination, (e.g. "I can´t stop thinking about how much it hurts"), magnification (e.g. "I´m afraid that something serious might happen") and helplessness (e.g. "There is nothing I can do to reduce the intensity of my pain") [29]. The PCS is a well-validated 13-item questionnaire and patients can answer on a 0-to-4 Likert scale (0 = “not at all” and 4 = “all the time”) [29]. The total score ranges between 0 and 52 and a total PCS score of 30 represents clinically relevant level of catastrophizing [29]. The higher the score, the more catastrophizing is present.

### Statistical analysis

Baseline and clinical characteristics are described as means with standard deviations (SD) in case of continuous variables and frequencies with accompanying proportions in case of categorical variables. The association of potential risk factors for pain (CSI, PCS, age, gender, BMI) at baseline and 12 weeks, and during follow-up (weeks) was assessed using linear regression analysis and mixed model analysis for repeated measures, respectively. Initially, univariate analyses were performed to identify potential risk factors. Factors that were significantly associated with the outcome (adjusted significance level of 0.10), were entered in a multivariate model (significance level 0.05). Adjustments for baseline values of the NRS were performed where appropriate. Fixed effects estimates with their 95% confidence intervals are presented (95% CI). A *p* value < 0.05 was considered statistically significant. No power analysis was performed since there were no data from previous studies to power on.

## Results

At baseline and 12-week follow-up, univariate analyses showed that gender and both the CSI and PCS were significantly associated with the NRS outcome (*p* ≤ 0.01). Multivariate analysis, however, revealed only the PCS as significantly being associated with the NRS with ß-values of 0.09 (*p* = 0.01) and 0.07 (*p* = 0.01), respectively (Table 2).

**Table 2:** Univariate and multivariate analyses of factors associated with the NRS pain at baseline, at 12 weeks and during follow-up (0–12 weeks)

Univariate analysis								
Baseline			At 12 weeks			0–12 weeks		
	ß (95%CI)	*p* value		ß (95%CI)	*p* value		ß (95%CI)	*p* value
						Follow-up (weeks)	−0.24 (−0.27 to −0.21)	< 0.01
Age	−0.13 (−0.09 to 0.07)	n.s	Age	0.02 (−0.02 to 0.06)	n.s	Age	0.01 (−0.04 to 0.05)	n.s
Gender	2.28 (0.88 to 3.68)	< 0.01	Gender	−0.12 (−0.95 to 0.70)	n.s	Gender	−0.80 (−1.64 to 0.03)	0.06
BMI	0.11 (−0.14 to 0.36)	n.s	BMI	0.01 (−0.13 to 0.14)	n.s	BMI	−0.03 (−0.17 to 0.11)	n.s
CSI	0.07 (0.03 to 0.12)	< 0.01	CSI	0.04 (0.01 to 0.06)	0.01	CSI	0.04 (0.01 to 0.07)	< 0.01
PCS	0.12 (0.06 to 0.18)	< 0.01	PCS	0.08 (0.05 to 0.12)	< 0.01	PCS	0.08 (0.05 to 0.12)	< 0.01
			NRS-BL	0.19 (0.04 to 0.34)	0.01	NRS-BL	0.32 (0.18 to 0.46)	< 0.01

Multivariate analysis								
Baseline*			At 12 weeks^#^			0–12 weeks		
	ß (95%CI)	*p* value		ß (95%CI)	*p* value		ß (95%CI)	*p* value
						Follow-up (weeks)	−0.25 (−0.28 to −0.22)	< 0.01
Gender	0.72 (−0.71 to 2.15)	n.s				Gender	−0.01 (−0.88 to 0.85)	n.s
CSI	0.04 (−0.01 to 0.08)	n.s	CSI	0.01 (−0.19 to 0.04)	n.s	CSI	0.01 (−0.02 to 0.04)	n.s
PCS	0.09 (0.02 to 0.16)	0.01	PCS	0.07 (0.02 to 0.11)	0.01	PCS	0.06 (0.01 to 0.10)	0.01
			NRS-BL	0.09 (−0.12 to 0.29)	n.s	NRS-BL	0.14 (−0.07 to 0.36)	n.s

*NRS* numeric rating scale, *CSI* central sensitization inventory, *PCS* pain catastrophizing scale, *NRS-BL* Numeric Rating Scale at baseline, *n.s* not significant

*r^2^ = *0.40*

^*#*^*r*^2^ = *0.44*

During 12-week follow-up, univariate analysis showed that gender, CSI, PCS were significantly associated with the NRS. Multivariate mixed model analysis showed that only the PCS remained significantly associated with the NRS outcome with a ß of 0.06 (95% CI 0.01–0.10, *p* = 0.01) (Table 2). Overall, decrease of the NRS at 12-week follow-up was 3.1 points (95% CI 2.4–3.8).

Twelve patients (32%) reported a CSI > 40 and three patients (8%) a PCS > 30 points.

**Fig. 1:**
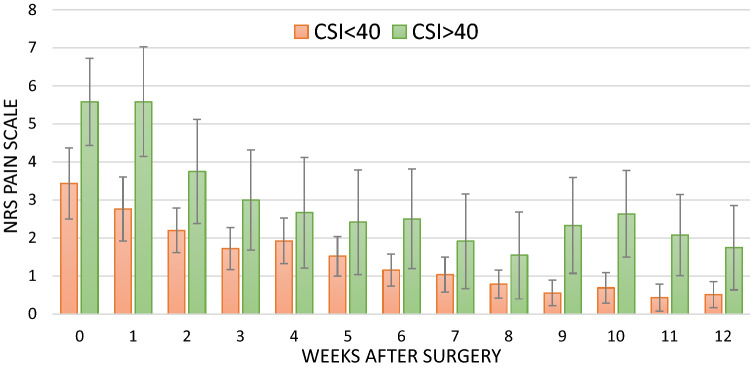
Mean (95% CI) NRS scores during 12-week follow-up stratified for the CSI (cut-off value 40). *NRS* numeric rating scale, *CSI* central sensitization inventory. Error bars represent 95% confidence interval

Additional univariate analysis using a cut-off value of 40 for the CSI showed that during 12-week follow-up, patients with a CSI > 40 scored on average 1,47 (95% CI 0.53–2.4) points higher on the NRS than patients with CSI < 40 (*p* < 0.01) (Fig. 1). This analysis was not performed for the dichotomized PCS as there were only three patients having a PCS > 30.

## Discussion

The most important finding of the present study is that the pain catastrophizing scale (PCS) was significantly associated with pain outcome at baseline, at 12 weeks as well as during 12-week follow-up in patients who had undergone hip arthroscopy. The central sensitization inventory (CSI) was only significantly associated with pain outcome after univariate analyses, and showed that patients with possible central sensitization, based on the cut-off value of 40 points, reported overall 1.5 points higher on the NRS compared to patients with a CSI < 40.

These results imply that the PCS was more strongly associated with pain outcome than the CSI. It is however debatable how much clinical significance the PCS has with only a ß of 0.06 meaning that for every point on the PCS patients reported 0.06 point higher on the NRS. The minimal clinical important change of the NRS is on average 1 point or 15% decrease in reported NRS [26].

There are some factors that are known to have a negative effect on the outcome after hip arthroscopy. These factors include increasing age, female gender and/or higher BMI [16, 28]. In our current study, we did not find any association between these factors and the outcome but this could have been caused because of the small sample size. Since no sample size calculation was performed for this study, the study could also be underpowered to detect the effect of the CSI. With respect to the dichotomized CSI, the difference of 1.47 points on the NRS with patients with CSI < 40 could indicate a clinically relevant effect [26]. As the variation of the PCS was too small to categorize patients as pain catastrophizing (*n* = 3), this analysis could not be performed for the PCS.

There is not much literature regarding the CSI and its effect on pain after hip arthroscopy. There are some papers that suggest a correlation between central sensitization and lower outcomes (or chronic pain) after total knee replacement surgery [18, 30]. In the paper of Jun Koh et al. the authors state that patients with pre-operative central sensitization show limited benefit of total knee arthroplasty compared to non-central sensitization patients [15].

A recent study by Dumont et al. shows that patients with FAIS and/or a diagnosis of depression or anxiety have higher levels on the pain catastrophizing scale [9]. In patients undergoing total knee arthroplasty, the level of pain catastrophizing is associated with higher postoperative pain, lower quality of life and lower patient reported outcomes after surgery [4, 14, 25]. Pain catastrophizing can be modified and is under influence of several factors, such as surgery, physical therapy, cognitive behavioral therapy and pharmacotherapy [11]. Surgical treatment itself can be a reason for a decrease in pain catastrophizing but it is important to realize that there is a group of patients that might benefit from pre-operative counseling, physiotherapy or even pharmacotherapy [11]. There is literature that shows a significant decrease in pain catastrophizing after a cognitive behavioral therapy program prior to orthopedic surgery [7]. These lower pre-operative pain catastrophizing scores resulted in lower postoperative pain and higher patient reported outcome scores after surgery [7].

Patient understanding and patient selection is important in the goal to achieve satisfying results after hip arthroscopy. The PCS and CSI may be of use in the pre-operative setting for measuring possible pain catastrophizing and identify those patients with high levels of pain catastrophizing or central sensitization. Both the PCS and CSI questionnaires are easy to use in daily practice and can give the orthopedic surgeon extra tools for identifying those patients that may benefit from pain catastrophizing reduction therapy (e.g. counseling) prior to surgery.

This study has some limitations: a small sample size, a small variation of the PCS and no results for the group of patients with a PCS > 30. The small sample size may have caused some instability in our results and a larger sample size would be necessary for correction of confounders to identify predictors for the NRS outcome.

## Conclusion

Results of this study show that both the PCS and the CSI are associated with the NRS reported pain at baseline and 12 weeks and during follow-up after hip arthroscopy. Unfortunately this study has a small sample size and future research is needed to detect if these results hold and whether treating central sensitization or pain catastrophizing might improve indications and outcome after surgery.
